# Inhibitory Actions of Tropeines on the α3 Glycine Receptor Function

**DOI:** 10.3389/fphar.2019.00331

**Published:** 2019-04-08

**Authors:** Victoria P. San Martín, Carlos F. Burgos, Ana M. Marileo, Cesar O. Lara, Anggelo Sazo, Jorge Fuentealba, Leonardo Guzmán, Patricio A. Castro, Luis G. Aguayo, Gustavo Moraga-Cid, Gonzalo E. Yévenes

**Affiliations:** Department of Physiology, Faculty of Biological Sciences, University of Concepción, Concepción, Chile

**Keywords:** glycine receptor, tropeines, modulation, ligand-gated ion channels, electrophysiology, molecular docking

## Abstract

Glycine receptors (GlyRs) are chloride-permeable pentameric ligand-gated ion channels. The inhibitory activity of GlyRs is essential for many physiological processes, such as motor control and respiration. In addition, several pathological states, such as hyperekplexia, epilepsy, and chronic pain, are associated with abnormal glycinergic inhibition. Recent studies have pointed out that positive allosteric modulators targeting the GlyR α3 subunit (α3GlyR) displayed beneficial effects in chronic pain models. Interestingly, previous electrophysiological studies have shown that tropeines, which are a family of synthetic antagonists of the serotonin type 3 receptors (5-HT_3_Rs), potentiate the activity of GlyRs conformed by α1 subunits. However, despite its importance as a pharmacological target in chronic pain, it is currently unknown whether the α3GlyR function is modulated by tropeines. Using electrophysiological techniques and molecular docking simulations, here we show that tropeines are inhibitors of the α3GlyR function. Tropisetron, a prototypical tropeine, exerted concentration-dependent inhibitory effects on α3GlyRs at the low micromolar range. In addition, three other tropeines showed similar effects. Single-channel recordings show that tropisetron inhibition is associated with a decrease in the open probability of the ion channel. Molecular docking assays suggest that tropeines preferentially bind to an agonist-free, closed state of the ion channel. The tropeine binding occurs in a discrete pocket around the vicinity of the orthosteric site within the extracellular domain of α3GlyR. Thus, our results describe the pharmacological modulation of tropeines on α3GlyRs. These findings may contribute to the development of GlyR-selective tropeine derivatives for basic and/or clinical applications.

## Introduction

Strychnine-sensitive glycine receptors (GlyRs) are anion-selective neurotransmitter-gated inhibitory ion channels. GlyRs belong to the pentameric ligand-gated ion channel superfamily, together with the inhibitory GABA_A_ receptor and the excitatory nicotinic acetylcholine receptor (nAchR) and serotonin type 3 receptors (5-HT3Rs) (Lynch, 2004; Lynch, 2009; Zeilhofer et al., 2012). In the mammalian CNS, the enhancement of the chloride conductance through GlyR activation results in a transient hyperpolarization of the membrane potential, which contributes to the control of neuronal excitability. The GlyR-mediated inhibition controls critical neurophysiological functions, such as motor coordination, respiratory control, muscle tone, as well as pain processing. Interestingly, several pathological states, such as hyperekplexia, epilepsy, autism, and chronic pain, have been associated with alterations on glycinergic inhibition (Harvey et al., 2004, 2008; Eichler et al., 2008; Pilorge et al., 2016).

Glycine receptors are pentameric complexes composed of α and β subunits, which can form receptors composed exclusively of α subunits (i.e., homomeric) or of α and β subunits (i.e., heteromeric) (Lynch, 2004; Corringer et al., 2012). Up until now, four α subunits (α1–4) and a single β subunit have been described. Although the α subunits share a high degree of sequence homology, they also exhibit important differences in their biophysical and pharmacological properties as well as in their expression patterns. These specific features are possibly linked to their proposed roles in physiological and pathological states (Lynch, 2009). A single GlyR subunit possesses an extracellular domain (ECD), four transmembrane domains (TM1-4) and an intracellular domain between the TM3 and TM4 domains (ICD). The binding of the neurotransmitter glycine to the ECD triggers the process of receptor activation, which results in the opening of the ion channel pore.

Despite the importance of GlyRs in physiological processes and in disease states, the present state of GlyR pharmacology is still limited (Yévenes and Zeilhofer, 2011; Zeilhofer et al., 2018). To date, only few agonists and antagonists of GlyRs have been characterized. Fortunately, recent studies have significantly expanded the GlyR pharmacology with the characterization of novel glycinergic positive allosteric modulators with analgesic actions in behavioral models of chronic pain (Zeilhofer et al., 2018). These studies pointed out that the potentiation of GlyRs composed of the α3 subunit is required for their *in vivo* analgesic actions. In this context, previous studies have shown that low nanomolar concentrations of several tropeines, which are a family of synthetic antagonists of the 5-HT_3_Rs (Haus et al., 2004), potentiate GlyRs composed of α1 subunits (Chesnoy-Marchais, 1996; Supplisson and Chesnoy-Marchais, 2000; Yang et al., 2007; Maksay et al., 2009). However, despite its importance as a pharmacological target in chronic pain, it is currently unknown whether the α3GlyR function is modulated by tropeines in a similar fashion. Defining the actions of this class of ligands on α3GlyRs will elucidate their pharmacological potential as chemical templates for the development of novel glycinergic modulators. Using electrophysiological techniques and molecular docking simulations, here we show that tropeines exerted inhibitory actions on α3GlyRs. In addition, our molecular docking studies suggest that tropeines likely bind to a discrete site near the glycine-binding site, preferentially in an agonist-free closed state of the ion channel.

## Materials and Methods

### Cell Culture and Transfection

HEK 293 cells (CRL-1573; American Type Culture Collection, Manassas, VA, United States) were cultured using standard methods (Lara et al., 2016). The cells were transfected using XfectTM Transfection Reagent (Clontech, United States) using 1.0 μg of cDNA plasmid encoding the α3GlyR subunit and 0.5 μg of EGFP. All recordings were made 24–36 h after transfection.

### Electrophysiology

Glycine-evoked currents were recorded from transfected HEK 293 cells in the whole-cell voltage-clamp configuration at room temperature (20–24°C) at a holding potential of -60 mV (Lara et al., 2016). Patch electrodes (3–4 mΩ) were pulled from borosilicate glass and were filled with (in mM): 120 CsCl, 8 EGTA, 10 HEPES (pH 7.4), 4 MgCl_2_, 0.5 GTP and 2 ATP. The external solution contained (in mM) 140 NaCl, 5.4 KCl, 2.0 CaCl_2_, 1.0 MgCl_2_, 10 HEPES (pH 7.4), and 10 glucose. Whole-cell recordings were performed with an Axoclamp 200B amplifier (Molecular Devices, United States) and acquired using Clampex 10.1 software. Data analysis was performed off-line using Clampfit 10.1 (Axon Instruments, Sunnyvale, CA, United States). Exogenous glycine-evoked currents were obtained using a manually applied pulse (3–4 s) of the agonist and an outlet tube (200 μm ID) of a custom-designed gravity-fed microperfusion system (flow rate ≈0.3–0.4 ml/min; exchange rate ≈50–80 ms) positioned 50–100 μm from the recorded cell. The methodologies employed for the single channel recordings of α3GlyRs in cell-attached configuration have been previously published (Marabelli et al., 2013; Lara et al., 2016). The patch pipettes (non-sylgard-coated) had tip resistances of 10–20 mΩ and were manually fire polished in a microforge (Narishige, Japan). The data were filtered (1-kHz low-pass 8-pole Butterworth) and acquired at 5–20 kHz using an Axopatch 200B amplifier and a 1322A Digidata (Axon Instruments, Union City, CA, United States). Data was acquired using pClamp software and analyzed off-line with Clampfit 10.1 (Axon Instruments, Union City, CA, United States). Tropeine stocks were prepared in high purity distilled water and subsequently diluted into the recording solution on the day of the experiment. Tropeines were obtained from AK Science (CA, United States). All other reagents were from Sigma-Aldrich (St. Louis, MO, United States).

### Molecular Docking Procedures

Protein-ligand docking was performed using the structures of α3 and α1GlyRs obtained from the Protein DataBank (PDB ID: 5CFB, 5TIO, 3JAD, and 3JAE) (Du et al., 2015; Huang et al., 2015). The structures of tropisetron, granisetron, dolasetron, and ondansetron are available in the PubChem database (CID: 656665, 3510, 3033818, and 4595) and were prepared using LigPrep before docking simulations. All complexes were created with Glide (Schrödinger, LLC, New York, NY, 2016) using a receptor grid centered on the amino acids that form the orthosteric binding site for glycine [(+)F159,Y202,T204,F207, (-)R65,S129, on α3GlyR], and an extra-precision (XP) configuration. Analysis of the interface GlyR-tropeines included structural and energetic parameters performed by the same software. To estimate ligand-binding affinity, a theoretical Gibbs free energy of binding, ΔG_bind_, was calculated by an energy calculation MM-GBSA using Prime (Schrödinger, LLC, New York, NY, 2016). All images were created using PyMol (v1.5, DeLano Scientific LLC, United States).

### Data Analysis

All values were expressed as mean ± s.e.m of normalized agonist-activated currents. *P* < 0.05 was considered statistically significant. Multiple comparisons were analyzed with ANOVA followed by a Bonferroni *post hoc* test. All the statistical analyses and plots were performed with MicroCal Origin 8.0 (Northampton, MA, United States).

## Results

**FIGURE 1:**
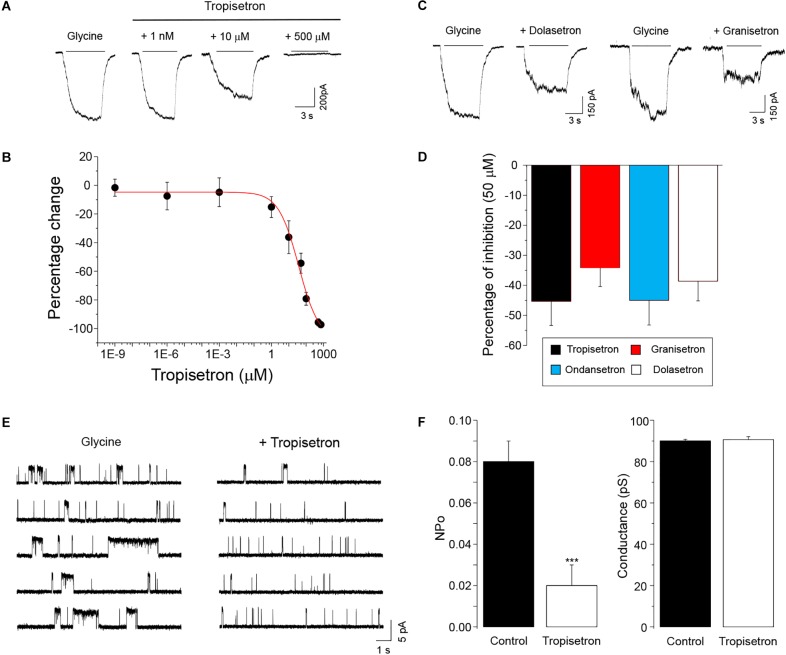
Functional modulation of homomeric α3GlyRs by tropeines. **(A)** Whole-cell current traces evoked by 30–60 μM glycine before and during the application of tropisetron (1 nM, 10 μM, and 500 μM) from a single HEK293 cell expressing α3GlyRs. **(B)** The graph summarizes the effect of different tropisetron concentrations (1 fM–700 μM) on the glycine-activated currents (*n* = 8). **(C)** Current traces evoked by 30–60 μM glycine before and after the application of 50 μM granisetron and dolasetron from two different HEK293 cells expressing α3GlyRs **(D)** The plot summarizes the effects of 50 μM granisetron (*n* = 6), ondansetron (*n* = 8), dolasetron (*n* = 7) and tropisetron (*n* = 6) on the glycine-activated currents of α3GlyRs. Differences were not significant (ANOVA followed by Bonferroni *post hoc* test, *F*(3,26) = 0.50). **(E)** Single-channel recordings in the cell-attached configuration from a cell expressing α3GlyRs before and in the presence of 50 μM of tropisetron. **(F)** The graphs show that tropisetron significantly decreased the open probability of α3GlyRs, but did not modify the main conductance (^∗∗∗^*P* < 0.001, paired Student *t*-test; *n* = 4).

We first examined the sensitivity of the homomeric α3GlyR to different concentrations of tropisetron (also known as ICS-205,930), a prototypical tropeine which is widely used as an anti-emetic drug. Application of tropisetron alone to cells expressing α3GlyRs did not elicit any detectable change in the holding currents, suggesting the absence of agonistic activity (not shown). Using a sub-saturating concentration of glycine (EC_5-10_) to activate GlyRs, we found that nanomolar concentrations of tropisetron did not modify the amplitude of the chloride currents through α3GlyRs (Figure 1A). On the other hand, concentrations of tropisetron above 1 μM exerted a significant inhibitory effect on the α3GlyR activity (Figure 1A,B). The concentration-response curve analysis displayed an IC_50_ of 37 ± 11 μM (*n* = 8), with a maximal inhibition of -97.2 ± 2.1% with 700 μM of tropisetron (*n* = 8). To obtain additional insights on the chemical determinants associated with the inhibition of α3GlyR by tropeines, we next analyzed the effects of three other tropeines on the α3GlyR function. We found that the application of 50 μM of granisetron, ondansetron, and dolasetron exerted a significant inhibition of the glycine-activated currents (Figure 1C,D). The extent of inhibition elicited by these compounds was not significantly different to those obtained with an equivalent concentration of tropisetron (Figure 1D), suggesting a conserved mechanism of inhibition. To investigate further the mechanism underlying the ion channel modulation, we next studied the tropeine modulation of α3GlyRs by performing single-channel recordings in the cell-attached configuration (Figure 1E,F). In agreement with our data obtained using whole-cell currents, the application of 50 μM of tropisetron to membrane patches expressing α3GlyRs significantly decreased the normalized open probability (nPo) by -76 ± 5.5%. On the other hand, tropeine did not elicit any significant change in the ion channel mean amplitude (Control = 5.40 ± 0.05 pA vs. tropisetron = 5.43 ± 0.09 pA, *p* = 0.65, paired *t*-test) or main conductance (Control = 90.0 ± 0.7 pS vs. tropisetron = 90.6 ± 1.5 pS, *p* = 0.59, paired *t*-test). Collectively, these results are consistent with a mechanism of ion channel inhibition resulting from the direct binding of tropisetron to the α3GlyR structure.

**FIGURE 2:**
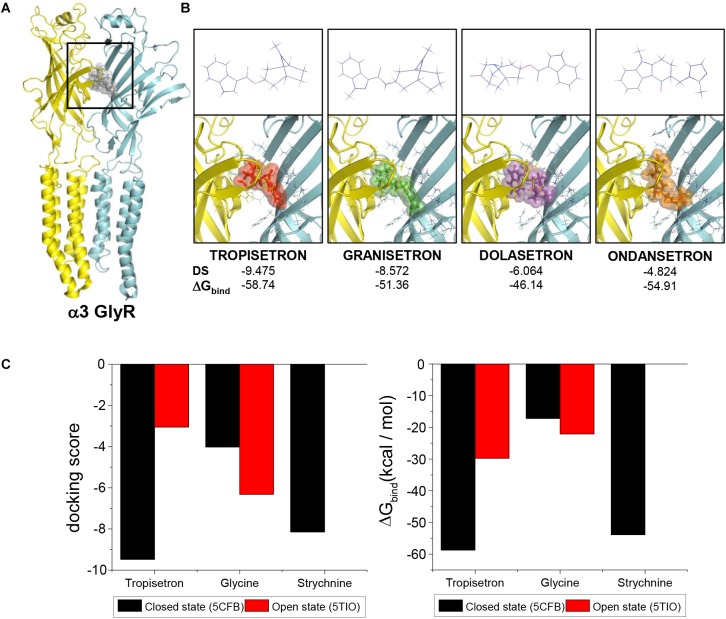
Putative binding sites of tropeines on the extracellular domain of α3GlyRs. **(A)** The α3/α3 dimer in closed conformation (from the 5CFB structure) shows the predicted binding site of the tropeines experimentally tested (gray spheres). Both protein chains are identical and were colored in cyan and yellow. **(B)** 2D structures (upper panels) and binding site on α3GlyR for tropisetron, granisetron, dolasetron, and ondansetron (lower panels). For all complexes, the side chains of the amino acids at 5Å around the modulator and forming part of the binding site are shown. Docking score and ΔG_bind_ values are presented for each compound. **(C)** The graphs compare the docking scores and the theoretical ΔG_bind_ of tropisetron interaction with α3GlyRs in the closed and in the open state. The values obtained from the docking procedures with glycine and strychnine under similar conditions has been added as references.

In order to explore the molecular and structural determinants of the tropeine inhibition, we next performed molecular docking assays using the crystal structures of α3GlyR as templates (Huang et al., 2015, 2017). All the tropeines tested were able to interact with the receptor in a favorable and stable manner (Figure 2A,B). A more detailed analysis of the binding site for tropeines showed that the amino acids S129, T204, and N42 of α3GlyR can form H-bonds with the modulators, while R65, F159, and F207 generate pi-cation interactions, and E157 forms a salt bridge with tropisetron (Supplementary Figure S1). Interestingly, the comparison between closed and open states suggests a significant preference of tropisetron to bind to the closed conformation of the ion channel (Docking scores, closed state: -9.475; open state: -3.055) (Figure 2B,C). In addition, an increase in the theoretical ΔG_bind_ was predicted for the open state complex (-58.74 to -29.75 kcal/mol) (Figure 2B,C). The results obtained with tropisetron were similar for the other tropeines examined (Supplementary Table S1).

## Discussion

The traditional view of GlyR pharmacology comprises a limited number of agonists, antagonists and allosteric modulators (Yévenes and Zeilhofer, 2011; Zeilhofer et al., 2018). An important part of these investigations centered their attention on the α1 subunit of GlyRs, which is widely expressed in the mammalian spinal cord and brainstem. However, due to its relevance in chronic inflammatory pain, the α3GlyR has attracted recent attention as a target for the development of novel analgesics. Several studies have demonstrated that novel compounds such as the propofol analog 2,6-di-tert-butyl phenol (2,6-DTBP), the phytocannabinoid cannabidiol (CBD), the natural alkaloid gelsemine, and the synthetic tricyclic sulfonamide AM-1488 are able to modulate glycine-activated currents through α3GlyRs (Xiong et al., 2012; Acuña et al., 2016; Lara et al., 2016; Huang et al., 2017; Zeilhofer et al., 2018). Interestingly, although none of these modulators possesses a significant GlyR subunit-selectivity, they were able to reduce chronic pain symptoms in behavioral models. The tropeines examined in our study, similar to many of these modulators, have also been characterized as modulators of other GlyR subunits. Several electrophysiological studies showed that tropisetron potentiated α1GlyR currents at nanomolar concentrations, while higher micromolar concentrations produced inhibition (Chesnoy-Marchais, 1996; Supplisson and Chesnoy-Marchais, 2000; Yang et al., 2007; Maksay et al., 2009). On the other hand, tropisetron only showed inhibitory effects on α2 GlyR activity (Supplisson and Chesnoy-Marchais, 2000). The results of the present work with α3GlyRs are similar to those obtained in studies with α2GlyRs and suggest that tropeines have a conserved mechanism of action on α2 and α3GlyRs. Interestingly, additional molecular modeling and docking simulations showed that the putative binding site of tropisetron is highly conserved between α1 and α3 GlyR subunits (Supplementary Figure S2), suggesting a similar drug-receptor interaction at least in a certain concentration range. Considering all of the above, it appears that the mechanisms underlying the divergence on the functional modulation elicited by tropisetron in α1GlyRs versus α2/α3GlyRs are linked to the allosteric transitions associated to the ion channel gating rather than differential binding modes of tropeines. Although the presence of more than one binding site of tropeines to GlyRs cannot be ruled out without further experimental evidence, we speculate that some GlyR configurations with very similar tropeine binding modes may trigger differential allosteric transitions, favoring a tropeine-dependent potentiation or a tropeine inhibition. Future studies may help to define a more comprehensive view of the mechanisms underlying the differential effects of tropeines on GlyRs of diverse composition.

## Conclusion

The present work describes the sensitivity of homomeric α3GlyRs to tropeines. Our results provide additional insights on the molecular mechanisms associated with the modulation of GlyRs by tropeines. A better understanding of the structural basis involved in the tropeine-GlyR interactions may contribute to the design of novel tropeine-based derivatives for basic and/or clinical applications.

## Author Contributions

VSM, CB, CL, AM, AS, and GM-C performed the research. JF, LG, PC, LA, GM-C, and GY designed the research and contributed with analytical tools. VSM, CB, CL, AM, GM-C, and GY analyzed the data. GM-C and GY wrote the manuscript. All authors read and approved the final version of the manuscript.

## Conflict of Interest Statement

The authors declare that the research was conducted in the absence of any commercial or financial relationships that could be construed as a potential conflict of interest.

## Supplementary Material

FIGURE S1 Ligand interaction diagrams of tropeines with the extracellular domain of α3GlyR. A detailed analysis of the amino acids involved in the formation and stabilization of α3GlyR complexes with **(A)** tropisetron, **(B)** granisetron, **(C)** dolasetron, and **(D)** ondansetron. All detected interactions are described in the inner box. All detected interactions between α3GlyR and each molecule are described in the inner box with a cutoff of 4Å from the receptor. Hydrogen bonds are partially electrostatic interactions between a hydrogen atom bound to an electronegative group and an atom or a group of atoms, which acts an acceptor. In the tropeine-α3GlyR interaction, S129, T204 act as hydrogen-bond donors, while the N42 acts as an acceptor. Pi-cation interactions are non-covalent interactions that occurs between a charged group with an electron-rich π group, like aromatic rings. Pi-cation interactions were obserbed between R65, F159, and F207 with tropisetron and granisetron. A salt bridge combines hydrogen bonds and ionic pairing. A salt bridge between the negative charged side chain of E157 with the NH2+ of several tropeines was observed. All analysis was performed using Maestro (Schrödinger, LLC, New York, NY, 2016).

FIGURE S2 Putative binding sites of tropeines on the extracellular domain of α1GlyRs. **(A)** The α1/α1 dimer in closed conformation (from the 3JAD structure) shows the predicted binding site for all tropeines tested (in gray spheres). Both chains are identical and were colored in light blue and pale yellow for representative purposes **(B)** 2D structure and binding sites of tropisetron, granisetron, dolasetron and ondansetron on α1GlyR. For all complexes, the side chains of the amino acids at 5Å around the modulator and forming part of the binding site are shown. Docking score and ΔG_bind_ are presented for each compound. ΔG_bind_ values were calculated using the equation *G* = E_bond_+E_electrostic_+E_vdW_+G_pol_+G_np_-TS, where G_pol_ corresponds to the solvation free energy obtained by using the generalized Born (GB) model, and Gnp is an estimation from solvent accessible surface area (SASA).

TABLE S1 Characterization of the complexes generated between the tropeines and α3GlyRs in closed / open state. Docking score and ΔG_bind_ calculated for complexes generated using the 5CFB (closed) and 5TIO (open) structures of α3GlyR. Values for glycine and strychnine obtained using a protocol similar to tropeines have been added as references.
