# Supplementary material for: Inhibitory Actions of Tropeines on the α3 Glycine Receptor Function
Source: Front Pharmacol. 2019 Apr 8;10:331. doi: 10.3389/fphar.2019.00331 (PMC6465966; doi:10.3389/fphar.2019.00331)
Supplement: FIGURE S1 — Ligand interaction diagrams of tropeines with the extracellular domain of α3GlyR. A detailed analysis of the amino acids involved in the formation and stabilization of α3GlyR complexes with (A) tropisetron, (B) granisetron, (C) dolasetron, and (D) ondansetron. All detected interactions are described in the inner box. All detected interactions between α3GlyR and each molecule are described in the inner box with a cutoff of 4Å from the receptor. Hydrogen bonds are partially electrostatic interactions between a hydrogen atom bound to an electronegative group and an atom or a group of atoms, which acts an acceptor. In the tropeine-α3GlyR interaction, S129, T204 act as hydrogen-bond donors, while the N42 acts as an acceptor. Pi-cation interactions are non-covalent interactions that occurs between a charged group with an electron-rich π group, like aromatic rings. Pi-cation interactions were obserbed between R65, F159, and F207 with tropisetron and granisetron. A salt bridge combines hydrogen bonds and ionic pairing. A salt bridge between the negative charged side chain of E157 with the NH2+ of several tropeines was observed. All analysis was performed using Maestro (Schrödinger, LLC, New York, NY, 2016). [file Data_Sheet_1.pdf]

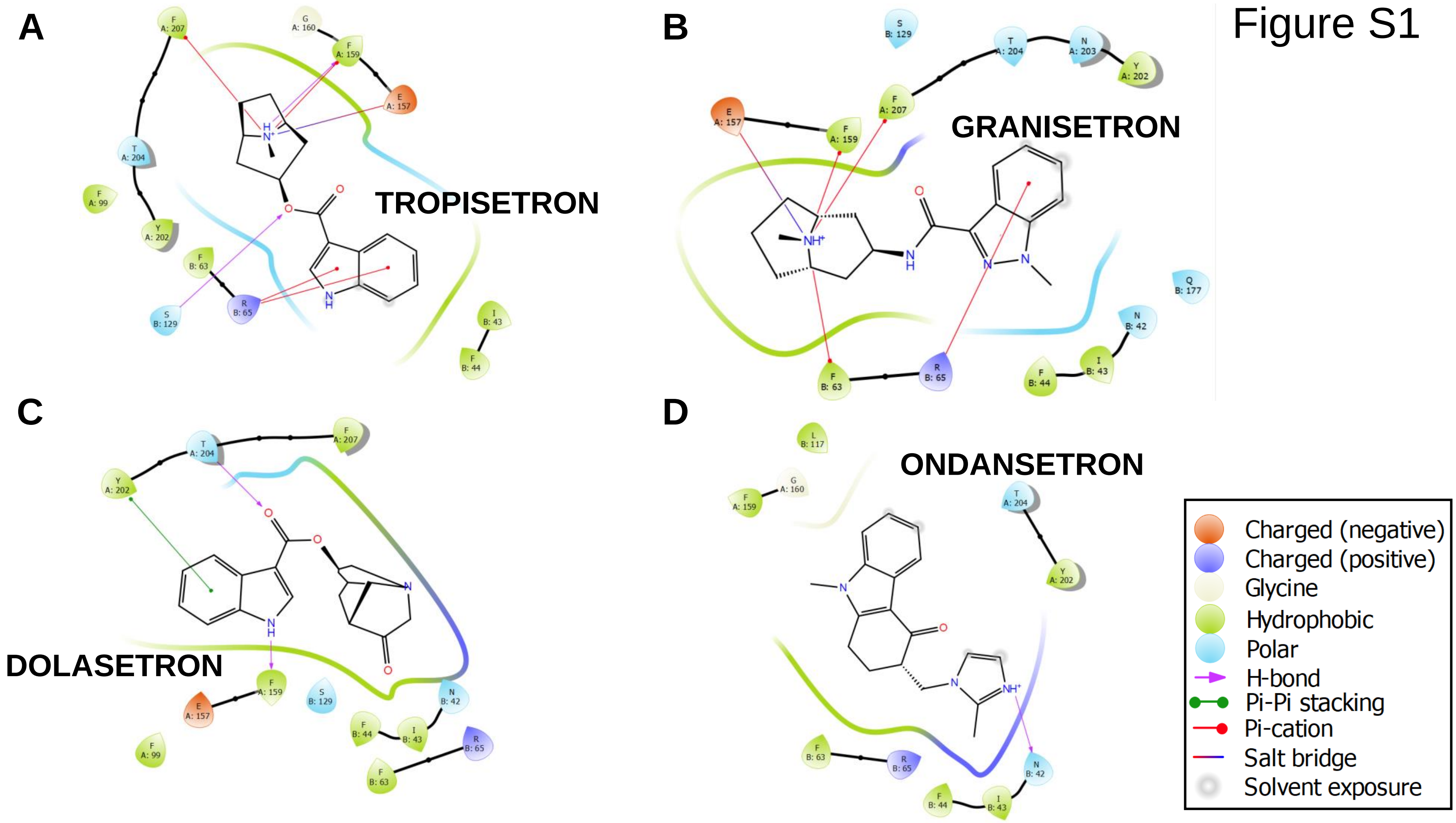
A
TROPISETRON
B
GRANISETRON
C
DOLASETRON
D
ONDANSETRON
Charged (negative)
Charged (positive)
Glycine
Hydrophobic
Polar
H-bond
Pi-Pi stacking
Pi-cation
Salt bridge
Solvent exposure

Figure S1

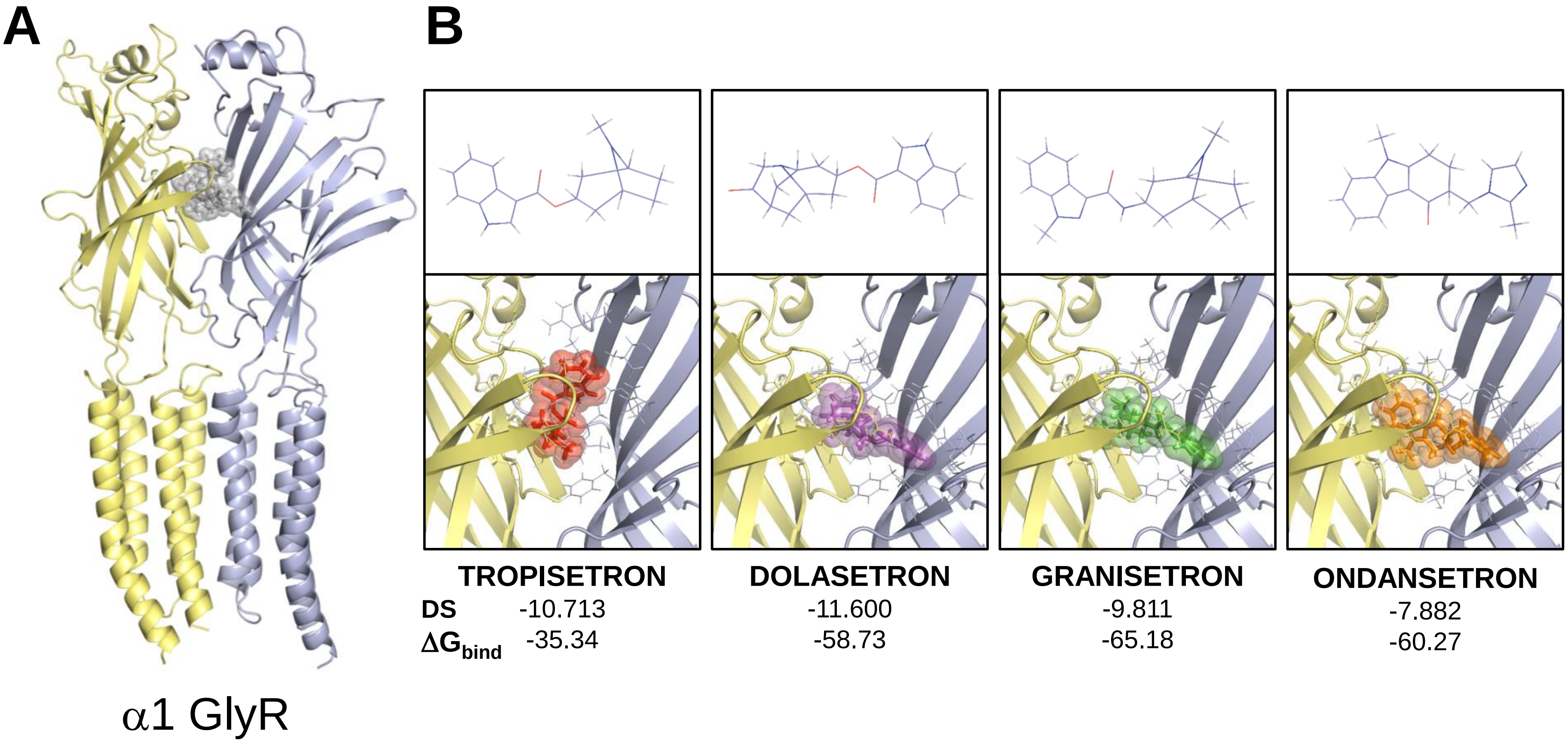
A
B
α1 GlyR
TROPISETRON
DOLASETRON
GRANISETRON
ONDANSETRON
DS -10.713 -11.600 -9.811 -7.882
ΔGbind -35.34 -58.73 -65.18 -60.27

Figure S2

	**Closed state** (5CFB)		**Open state** (5TIO)	
	Docking score	ΔG_{bind} (kcal/mol)	Docking score	ΔG_{bind} (kcal/mol)
Tropisetron	-9.475	-58.74	-3.055	-29.75
Granisetron	-8.572	-51.36	NC	NC
Dolasetron	-6.064	-46.14	-4.144	-41.14
Ondansetron	-4.824	-54.92	-3.665	-40.97
Glycine	-4.021	-17.20	-6.321	-22.07
Strychnine	-8.149	-53.90	NC	NC

NC: no complex was formed

Table S1
